# Artifact identification in X-ray diffraction data using machine learning methods

**DOI:** 10.1107/S1600577522011274

**Published:** 2023-01-01

**Authors:** Howard Yanxon, James Weng, Hannah Parraga, Wenqian Xu, Uta Ruett, Nicholas Schwarz

**Affiliations:** a Argonne National Laboratory, 9700 South Cass Avenue, Lemont, IL 60439, USA; Advanced Photon Source, USA

**Keywords:** *in situ* synchrotron high-energy X-ray powder diffraction, machine learning, image identification and recognition

## Abstract

*In situ* synchrotron high-energy X-ray powder diffraction (XRD) is highly utilized by researchers to analyze the crystallographic structures of materials in functional devices (*e.g.* battery materials) or in complex sample environments (*e.g.* diamond anvil cells or syntheses reactors). An atomic structure of a material can be identified by its diffraction pattern along with a detailed analysis of the Rietveld refinement which yields rich information on the structure and the material, such as crystallite size, microstrain and defects. For *in situ* experiments, a series of XRD images is usually collected on the same sample under different conditions (*e.g.* adiabatic conditions) yielding different states of matter, or is simply collected continuously as a function of time to track the change of a sample during a chemical or physical process. *In situ* experiments are usually performed with area detectors and collect images composed of diffraction patterns. For an ideal powder, the diffraction pattern should be a series of concentric Debye–Scherrer rings with evenly distributed intensities in each ring. For a realistic sample, one may observe different characteristics other than the typical ring pattern, such as textures or preferred orientations and single-crystal diffraction spots. Textures or preferred orientations usually have several parts of a ring that are more intense than the rest, whereas single-crystal diffraction spots are localized intense spots owing to diffraction of large crystals, typically >10 µm. In this work, an investigation of machine learning methods is presented for fast and reliable identification and separation of the single-crystal diffraction spots in XRD images. The exclusion of artifacts during an XRD image integration process allows a precise analysis of the powder diffraction rings of interest. When it is trained with small subsets of highly diverse datasets, the gradient boosting method can consistently produce high-accuracy results. The method dramatically decreases the amount of time spent identifying and separating single-crystal diffraction spots in comparison with the conventional method.

## Abstract 2

The capability of machine learning methods for identifying and separating artifacts that appear in a typical X-ray diffraction image is demonstrated.

## 1. Introduction

X-ray user facilities produce some of the largest scientific datasets in the world. The experiments performed at these facilities cover a large range of multi-disciplinary sciences and produce data which are often the sum of various materials in the path of the X-ray beam. The identification of the data of interest, assurance of sufficient data quality and recognition of systematic errors require a high level of expertise. It is therefore essential to coordinate data collection and analysis to achieve optimized efforts in scientific research. Many endeavors have been made to optimize data acquisition processes to improve scientific reproducibility, transparency and reliability, overcoming limitations in data quality to open pathways to new discoveries. The success of these efforts will enable users to efficiently search and effectively comprehend the necessary measures for understanding the complex concepts of natural phenomena.

Artificial intelligence (AI) and machine learning (ML) advances have shown promise, not only in speeding up, but also expanding the robustness of data analysis methods, and are poised to play an important role in X-ray user facilities (Sivaraman *et al.*, 2021*a* ▸,*b* ▸; Du *et al.*, 2020 ▸; Cherukara *et al.*, 2018 ▸; Liu *et al.*, 2020 ▸, 2022 ▸; Yang *et al.*, 2018 ▸). For example, *PtychoNN* – a Python library that leverages deep convolutional neural networks (CNNs) to solve image reconstruction problems in ptychograpic X-ray imaging – has improved the speed by two orders of magnitude and with five times less data than required by current methods which use phase retrieval algorithms (Cherukara *et al.*, 2020 ▸). An approach that uses an ML clustering method to remove spurious data in Bragg coherent diffraction imaging has decreased the amount of time spent in manual data processing (Pelzer *et al.*, 2021 ▸). Additionally, grazing-incidence small-angle X-ray scattering (GISAXS) is a surface-sensitive technique that has seen a tremendous growth in popularity in probing complex morphologies ranging from the fields of polymer and soft-matter science to hard condensed matter (Hexemer & Müller-Buschbaum, 2015 ▸). The scattering patterns can hold a variety of features, such as peaks, rods and rings, whose position in reciprocal space can often express its real space features. Several use cases of ML techniques have been successfully applied to other studies similar to the GISAXS type of problem (Wang *et al.*, 2017 ▸; Kiapour *et al.*, 2014 ▸; Ziletti *et al.*, 2018 ▸). Another example of ML application is illustrated in the post-data analysis of X-ray diffraction (XRD) experiments. ML methods have been developed and compared to classify crystal structure symmetries and space groups from simulated and experimental 1D XRD patterns, resulting in interpretable outcomes (Oviedo *et al.*, 2019 ▸; Suzuki *et al.*, 2020 ▸). Deep variational autoencoders applied to simulated and experimental 1D XRD patterns can be used for classification and XRD reconstructions in a Co–Cr–Ni–Re system composed of three phase mixtures (Banko *et al.*, 2021 ▸). Finally, phase maps of an Fe–Co–Ni ternary alloy can be constructed by measuring the dissimilarity among the XRD patterns (Iwasaki *et al.*, 2017 ▸).

Recent advances in ML techniques have had tremendous impact across industry and scientific domains, especially in the application of image processing. For instance, CNN and gradient boosting are successfully applied and used in image segmentation (Badrinarayanan *et al.*, 2017 ▸; Chen *et al.*, 2014 ▸; Yu & Koltun, 2015 ▸) and image recognition (Simonyan & Zisserman, 2014 ▸; He *et al.*, 2016 ▸; Peter *et al.*, 2017 ▸). These methods require diverse datasets on which the algorithms can train to select values through potentially millions of hyperparameters required to produce accurate models. The datasets are necessary to provide sufficient samplings of the relevant feature spaces. The ML recognition and segmentation applications can be carried over to other datasets such as XRD images.

In this paper, we demonstrate the capability of ML methods for recognizing and segmenting artifacts that appear in a typical XRD image. The *in situ* synchrotron high-energy XRD technique (Ren, 2012 ▸) is widely used to study the crystallographic structures of inorganic materials. A crystallographic structure of a material can be identified by its diffraction pattern along with a detailed analysis of the Rietveld refinement which yields rich information on the structure and the materials, such as crystallite size, microstrain and defects. XRD images/patterns can be collected under different conditions such as pressure, temperature, electrical field and/or gas atmosphere, which can yield different states of matter. Since the Advance Photon Source (APS) has a plethora of XRD images collected by many user groups, XRD experiments are well suited to benefit by exploiting ML methods.

XRD images are usually composed of many characteristics such as rings, preferred orientations or texture, and single-crystal diffraction spots. These characteristics can be identified by their positions, shapes and intensities. For example, a single-crystal diffraction spot is usually a localized spot round in shape or close to a round shape, spanning a few pixels to a few tens of pixels in diameter, with very high intensity. In many situations, it is desirable to separate single-crystal diffraction spots before the integration procedure in order to produce the conventional 1D XRD pattern with accurate peak intensities and profiles. *GSAS-II* (Toby & Von Dreele, 2013 ▸) – state-of-the-art software for crystallographic structural analysis – has a segmentation algorithm that can automatically identify single-crystal diffraction spots based on image pixel intensity. Nevertheless, the algorithm can fail to differentiate the artifacts when other characteristics such as preferred orientations are also present in an XRD image.

The performance of several ML methods is compared based on their abilities to mask out single-crystal diffraction spots. The demonstration shows that ML methods are suitable approaches to recognize and separate (*i.e.* mask) both speed and accuracy despite a number of confounding factors that may impact the accuracy of results, such as peak shifting due to lattice expansion or contraction. In addition, the speed-up makes ML methods applicable for on-the-fly masking during an XRD experiment. This implementation not only reduces the person power required to analyze data but also enables correct on-the-fly data analysis to optimize data collection.

## 2. Data and methods

### 2.1. Datasets

**Figure 1:**
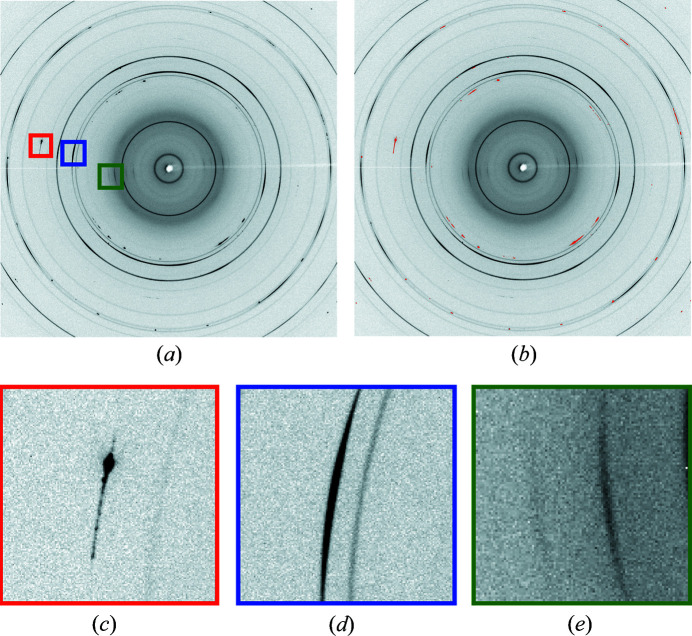
(*a*) Raw experimental XRD pattern and (*b*) its masking result. The red, blue and green boxes in (*a*) show (*c*) single-crystal diffraction spots, (*d*) preferred orientation (the darker line) and two texture lines. Panels (*a*) and (*b*) are cropped for visualization purposes.

For this study, we used five diverse datasets comprising raw XRD images belonging to different material compositions. The datasets are collected at the APS beamline 17-BM (see Table 1 ▸). The raw images are 2880 × 2880 pixel intensity arrays, collected with a Varex XRD 4343CT area detector. As mentioned above, an XRD image can contain different characteristics as observed in Fig. 1 ▸. In the figure, the intensities of the preferred orientation and single-crystal diffraction spots are often the same order of magnitude. The preferred orientations show differential intensities around a diffraction ring and are usually symmetric. This means that an intense band at a particular azimuth angle is met with a similarly intense band in the opposite or 180° direction, whereas the single-crystal diffraction spots can be attributed to scattering from the sample holder, single-crystal phases formed in a reaction *etc.* Each dataset is obtained under unrelated experimental conditions, such as different wavelength, detector center, sample-to-detector distance *etc.* The information is stored as metadata along with the detector image. Meanwhile, the overall scale of pixel intensities in an XRD image is related to the inlet photon flux of the beam, the amount of sample in the beam path, the sample scattering power, the total exposure time and the detector gain setting.

The Nickel dataset contains 11 images of powder diffraction rings with single-crystal diffraction spots spreading throughout the patterns. The purpose of this dataset is to provide a benchmark in accuracy and time performance of the ML algorithms used in this study. Since the images contain many single-crystal diffraction spots, the dataset is ideal for inspecting different ML algorithms. In order to generate training and testing sets, the Auto Spot Mask (ASM) search of the *GSAS-II* software is employed as it is well suited for single-crystal diffraction-spot detection.

*GSAS-II* (Toby & Von Dreele, 2013 ▸) is a popular crystallography data analysis software application written in the Python programming language. Prior to determining the crystal structure of a material, *GSAS-II* enables users to remove irrelevant signals (*e.g.* single-crystal diffraction spots) from XRD images. This is facilitated by the *GSAS-II* ASM search capability. The ASM method checks one thin shell (expanded in the radial direction) at a time until it reaches the maximum 2θ value used for integration, which is typically set close to the edge of the detector. Namely, the corners of the image are often ignored since it cannot make a circular thin shell without being trimmed. Once a thin shell is specified, the search will gather all of the pixel intensities within the thin shell and disregard the intensities that are larger than *F*(ε) = 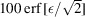 , where ε is a user-defined hyperparameter with the range 1–10. A smaller value of ε indicates more aggressive masking. Additionally, erf is the error function. The standard deviation (σ) is calculated based on the intensities that are smaller or equal to *F*(ε). Subsequently, the ASM search will mask the pixels (**x**) based on the condition 

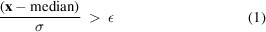

where the median intensity is calculated without neglecting intensities larger than *F*(ε).

Although the ASM search can detect the single-crystal diffraction spots very well, it often fails to exclude preferred orientations or textures in the masking process. This shortcoming is because these characteristics also have high intensity, skewing the distribution of pixels within the thin shell further from the normal. In contrast to the spots, preferred orientations are desired signals in the 2D XRD pattern that should be included in the integration process. Another disadvantage of using the ASM search is that the computational speed is relatively slow. It takes roughly 230 s to process one XRD image on a workstation with an Intel Xeon Silver 4110 2.10 GHz 32 core CPU. Accordingly, the expensive portion of the code can be re-written using the C Foreign Function Interface (CFFI) (Rigo & Fijalkowski, 2018 ▸) Python library. By employing the C programming language to process the highly time-consuming portion of the code, the computation time is reduced by 96% to around 8.8 s per image on average. This improvement in speed may help to potentially enable XRD data processing on-the-fly during an experiment. Nevertheless, a speedup by an order of magnitude can be achieved by ML methods, as will be shown later.

The Battery-1 dataset contains 11 XRD images with single-crystal diffraction spots and preferred orientations or textures. The dataset is certainly more complex than the Nickel dataset as multiple characteristics are present. Due to the charging/discharging nature of the experiment, distinct patterns can be traced in each of the images since each pattern may embody different signals from the charge carrier. Battery-2, Battery-3 and Battery-4 also are associated with the charging/discharging experimental conditions, containing single-crystal diffraction spots and textures. Manual masking is carefully implemented for the single-crystal diffraction spots to create the training and test sets for these datasets as the ASM search can falsely identify proper signals.

Each dataset represents a different chemical composition and is collected under independent experimental conditions, ensuring variability in the XRD patterns. Accordingly, each image in a dataset is collected with the same wavelength, detector center, distance to detector *etc.* Depending on the nature of an experiment, every image from a dataset may possesses different peak positions in accordance with the 2θ axis. For example, peaks in a battery system can notably shift due to the migrations of the charge carriers in a charging/discharging experiment. Meanwhile, peaks can shift due to lattice expansion in temperature-ramping experiments.

### 2.2. Feature engineering

**Figure 2:**
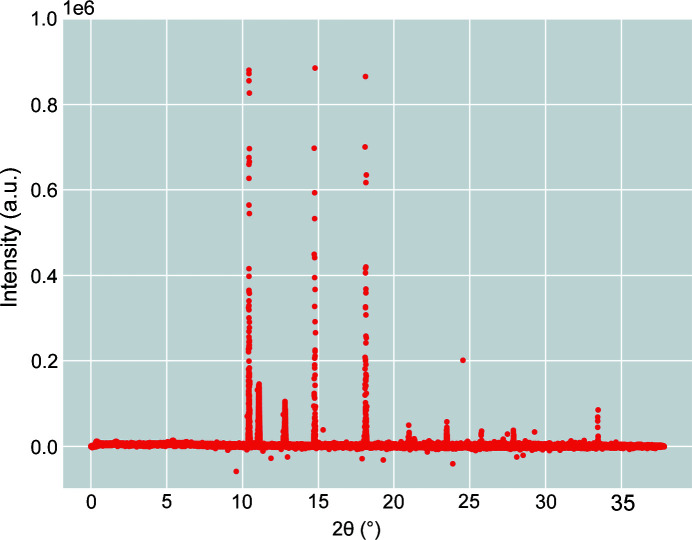
Plot illustrating the feature space of the 2D XRD pattern shown in Fig. 1 ▸ in which every point belongs to a pixel.

The feature engineering process manipulates and transforms raw data into features prior to ML training. The features act as the input values used by the ML method in order to improve its performance (Zheng & Casari, 2018 ▸). Aside from the intensity of an XRD image, features such as 2θ and azimuth angles and pixel locations are utilized to enhance ML performance. Both 2D 2θ and azimuth maps are constructed by converting the metadata (*e.g.* wavelength, detector center, distance to detector *etc.*) geometrically. Fig. 2 ▸ depicts the intensity–2θ feature space. Sets of features are defined in Table 2 ▸ for convenience in the later discussion.

Furthermore, a standard scaling normalization (Raju *et al.*, 2020 ▸) routine will be applied to all the features prior to ML training,

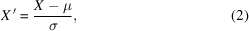

where μ and σ are the mean and the standard deviation of the feature values (*X*), respectively. The normalization routine offers robustness to an ML model since the intensity values can be an order-of-magnitude higher than the other features’ values. It can also provide numerical stability, such as avoiding vanishing/exploding gradients in ML training if a normalization schema is appropriately implemented within an ML pipeline (Ioffe & Szegedy, 2015 ▸).

### 2.3. Machine learning methods

Support vector machines (SVM) (Cortes & Vapnik, 1995 ▸), *k* nearest neighbors (KNN) (Peterson, 2009 ▸), extra trees, random forest (Ho, 1995 ▸) and gradient boosting (Friedman, 2001 ▸) algorithms are compared using experimental XRD images. The SVM algorithm separates classes of labels by determining the decision boundaries, whereas the KNN technique is a voting algorithm that examines the *n*-closest neighbors to a data point and decides a class label based on its neighbors. The extra trees, random forest and gradient boosting algorithms belong to the ensemble classification family. An ensemble algorithm typically generates many distinct models and averages the models to create more refined predictive results. In terms of model complexity, the ensemble algorithms are more complex than the SVM and KNN algorithms. Hence, they are expected to yield better predictive results in general.

*scikit-learn* (Pedregosa *et al.*, 2011 ▸) and *XGBoost* (Chen *et al.*, 2015 ▸; Chen & Guestrin, 2016 ▸) libraries are employed for ML applications. *scikit-learn* – a Python library for ML applications – is utilized to employ the SVM, KNN, extra trees and random forest algorithms. Multi-core CPU implementations are available in *scikit-learn* for all these methods with the exception of the SVM method. Since the Intel Xeon Silver 4110 2.10 GHz CPU used in this work has 32 cores, 32 CPU cores will be exploited for all training and testing procedures, apart from training and testing using the SVM method. The *XGBoost* library is used for the gradient boosting framework. The *XGBoost* library allows the user to exploit multi-core CPUs as well as a graphical processing unit (GPU). A GeForce RTX 2080 Ti GPU is used in this research. Results using both a multi-core CPU and a GPU will be evaluated and compared in terms of time performance.

All the ML methods require input values and return a class label as the output for every data point. There are 2880 × 2880 = 8 294 400 data points in one XRD image. Consequently, the ML algorithms will produce a 2D mask map with 8 294 400 outputs, yielding two label classes: 0 (no mask) and 1 (mask). For the input values, intensity, angles and pixel locations are utilized as mentioned in Section 2.2. In Section 3.1, the intensity and 2θ features are chosen as the input values while inspecting and comparing the five ML methods using the Nickel dataset.

The accuracy and time performances will be evaluated among the five ML algorithms using the Nickel dataset. The comparison reveals promising algorithms that can handle experimental XRD images. For accuracy, fivefold cross validation will be applied within a grid search to find the optimal hyperparameters for each of the ML algorithms. Three random raw unique images are selected as a training dataset, and the remaining images are used as the test dataset for every fold. The averages of accuracy and time are applied within the fivefold cross validation. The accuracy performance is measured by the recall and specificity scores, 

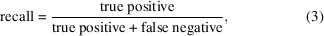




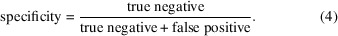

The recall (true positive or TP rate) score can be considered to be the ability of an ML classifier to find all the masked pixels. The specificity (true negative or TN rate) is also shown, but not as an accuracy evaluation metric. When there is a label class imbalance [see Fig. 1 ▸(*b*)], the TP rate emphasizes the accuracy on the infrequent class, and the TN rate characterizes the classifier performance over the larger negative class. Thus, a natural choice for accuracy is the recall or TP rate.

After juxtaposing the five ML methods, the trained gradient boosting model will be employed to predict the Battery-1 dataset. This assessment will expose the transferability of the trained model to a more complicated dataset, consisting of several characteristics. As the Battery-1 dataset has highly contrasting characteristics, it is expected that the trained model will perform poorly, since ML algorithms are known to perform well for interpolation but poorly for extrapolation. As shown in the Section 3.2, the trained model can be rectified by including a small subset of the Battery-1 dataset and the feature engineering approach to attain better predictive results. The feature engineering approach involves inclusion of the azimuth angle and pixel locations, which improves the performance of the gradient boosting model significantly.

**Figure 3:**
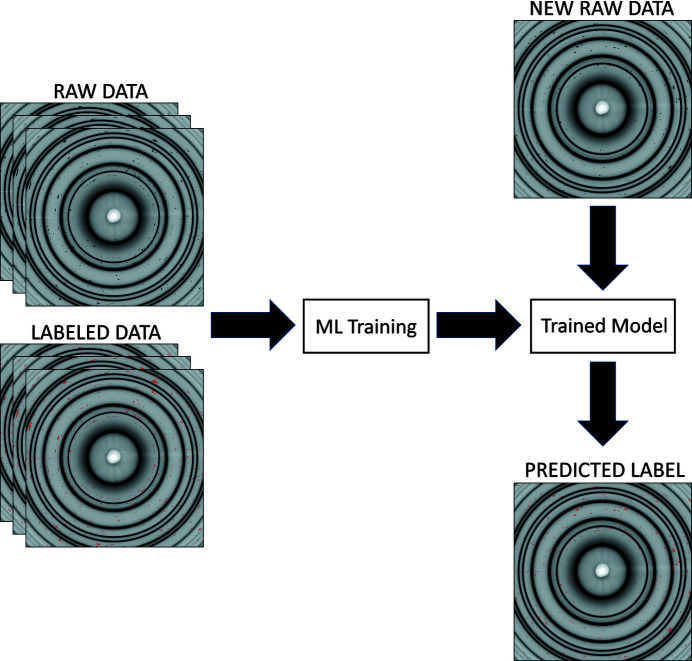
Schematic of the ML pipeline that can be used at a beamline for identifying single-crystal diffraction spots. A few images will be collected and manually labeled or more likely using ASM search prior to ML training. The rest of the data will be labeled using the trained model.

Lastly, we explore the predictive ability of the gradient boosting algorithm against all the datasets, providing diversity in chemical compositions, characteristics and experimental conditions. In this case the Nickel, Battery-1, Battery-2, Battery-3 and Battery-4 datasets are used to assess the ML algorithms. Three images will be selected at random from each dataset as the training set, while the remaining images are considered the testing set, providing wide diversity in the training and testing sets. The process will discover the model adaptability against various types of XRD images, belonging to different materials and collected under different experimental conditions. A schematic of the ML pipeline used in this study is shown in Fig. 3 ▸.

## 3. Results and discussion

### 3.1. Machine learning algorithm assessment

Grid searches with fivefold cross validations using the Nickel dataset are applied to tune hyperparameters for the SVM, KNN, extra trees, random forest and gradient boosting methods. For every onefold cross validation, three random unique images are selected as the training set, while the remaining images serve as the test set. We ideally prefer to use as few images as possible (*i.e.* one labeled image = 8 294 400 data points) as the training dataset. The Nickel dataset showed no significant improvement in accuracy with more than three images or >24 million data points included in the training. The optimal hyperparameters, obtained by executing the fivefold cross validation, are presented in Table 3 ▸. Most of the hyperparameter values lie on the aggressive side. However, there is no indication of overfitting according to the predicted results. Table 4 ▸ summarizes the accuracy and time performances of the ML methods.

The SVM performances suggest that the method is unsuitable for this type of data. The method cannot find convergence given the training dataset. The training is set to terminate after 10 000 iterations. The algorithm fails to find the boundary decision given the complex feature space (*i.e.* the feature space contains spikes of intensities). One can use a high-degree polynomial function as the kernel, instead of the radial basis function. However, this can lead to overfitting. Moreover, the prediction time takes orders of magnitude longer than the other ML methods and the ASM search.

The KNN results are more encouraging. As mentioned above, the KNN algorithm predicts a new data point based on its neighboring data points. Consequently, it is preferable to choose an odd number of *k* neighbors so that the algorithm can conclusively decide when there is an equal number of positive and negative votes. The TP and TN rates suggest that the KNN algorithm can produce reliable predictive results in comparison with the SVM method. The high TN rate shows that the method can sufficiently predict negatively labeled pixels. Meanwhile, the TP rate displays an adequate prediction, yielding 94.69% accuracy despite the class imbalance. In spite of the reliable predictive capability, the time performance takes four times longer than the *GSAS-II* ASM search powered with the CFFI module. In this case, the predictive time shows that the algorithm is not ideal for on-the-fly masking in an XRD experiment. In addition, the predictive time of the algorithm is a function of the training dataset. This prediction time of the model will increase as the size of the training dataset increases.

The ensemble algorithms show better predictive results in which the gradient boosting framework is the best of all the ML methods studied in this research. The extra tree method is able to capture the TP more effectively than the KNN algorithm. However, the TN rate seems to perform slightly worse than the KNN algorithm. Nevertheless, the prediction time is greatly reduced by one order of magnitude, which is twice as fast as the *GSAS-II* ASM search with the CFFI module. The training time takes as long as the KNN algorithm. There is no distinct difference in TP rates between the extra tree and random forest methods. However, the random forest method takes longer to train on average, whereas the prediction time performance is the same for the two methods.

The gradient boosting framework seems to perform the best in terms of the TP rate and time performance. Note that the gradient boosting technique is the superior algorithm compared with the other methods in this study, so it is expected to outperform the others. The training time in a multi-core CPU and in a GPU are 2.2 and 0.94 s, respectively. The time performances are superior to the rest of the algorithms and the ASM search. Even with multi-core usage, the training time takes an order of magnitude less time than the other ML methods. If training time is not an issue, the gradient boosting framework can potentially be trained with many different types of raw XRD images collected from different experiments to further enhance the predictive power of the model. Furthermore, the prediction time of the gradient boosting method (or the other ensemble methods) does not depend on the number of training points, so the prediction time remains the same as the training dataset size becomes larger. If a GPU is available, the gradient boosting framework can achieve the most efficient on-the-fly masking for an XRD experiment compared with the other algorithms in this study.

### 3.2. Feature assessment

We will inspect the transferability of the gradient boosting model, where the training procedure is described in Section 3.1 except that the n_estimators parameter is increased to 100. The trained model will be used to evaluate the Battery-1 dataset. Compared with the Nickel dataset, the Battery-1 dataset is more complex, as the images contain preferred orientations or textures, and single-crystal diffraction spots. The *GSAS-II* ASM search is unable to distinguish among these characteristics. The previously trained gradient boosting model (*i.e.* only the Nickel dataset is included as a training dataset) also performs poorly in predicting the Battery-1 dataset. The poor prediction is expected as ML methods can reasonably interpolate data but poorly extrapolate data. Since the Battery-1 dataset was excluded in the previous gradient boosting training, the model predicts the overall TN and TP rates to be 97.3 and 17.0%, respectively.

**Figure 4:**
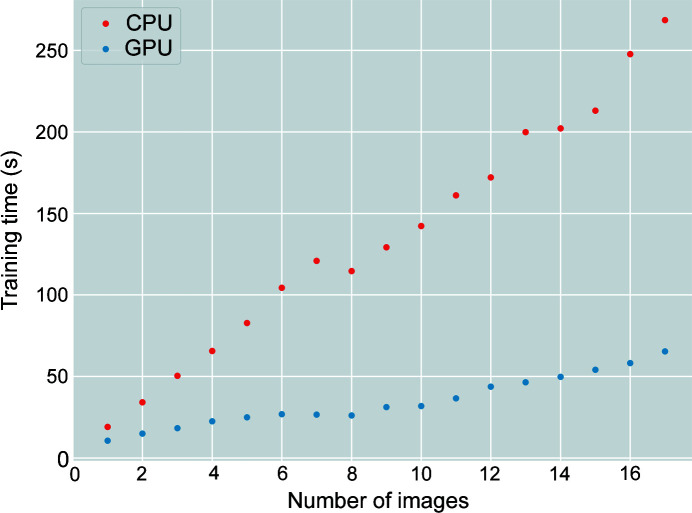
Training time performances as a function of the number of training images based on the gradient boosting algorithm.

To increase the predicted TP rate of the Battery-1 dataset, a small subset is included in the training of the gradient boosting model. After including three images selected at random from the Battery-1 dataset along with the three random images from the Nickel dataset, the predicted TP rate of the Nickel dataset undergoes a modest reduction to 95.4%, and the TN rate remains the same at 99.9%. The training time with a multi-core CPU increases to 79 s, whereas the prediction time per image increases to 3.8 s. The training time with the GPU increases to 20 s, whereas the prediction time per image remains roughly unchanged at 0.93 s. Meanwhile, the overall TN and TP rates increase to 100.0 and 54.1%, respectively. More images of the Battery-1 dataset are included in the training as an attempt to increase the TP rate. At the maximum number of images, the TP rate increases to only 54.4% as the training time increases linearly as a function of the number of images (see Fig. 4 ▸). The low TP rate of the Battery-1 dataset could be due to the fact that the images embody more complex characteristics that require a more powerful model to be able to generate finer results.

Furthermore, feature engineering is used to strengthen the gradient boosting model by incorporating the azimuth angle and/or pixel locations as features. Again, three images are selected at random from each of the Nickel and Battery-1 datasets. The TN rates for both datasets remain at 100.0%. The overall predicted TP rates of Battery-1 increases remarkably as shown in Table 5 ▸. In general, the overall predicted TP rates improve as the number of features increases for both Nickel and Battery-1 datasets. The TP rate for the Nickel dataset increases from 95.4% (two features) to 98.9% (five features), and the TP rate for Battery-1 experiences a prominent improvement from 54.8% (two features) to 98.0% (five features). Adding the azimuth angle or pixel locations boosts the accuracy by 30.6 or 42.7%, respectively, while a slight improvement of 0.5% is observed using feature set D in comparison with feature set C for the Battery-1 dataset. The lower improvement in accuracy of using the azimuth angle as a feature compared with the locations can be caused by the location feature which contains two orthogonal descriptions such as *x* and *y* pixel positions.

The time performances increase slightly. As for GPU time performance, the training time shows a slight increase from 20 s (two features) to 25 s (five features), whereas the prediction time per image shows a slight increase from 0.93 s (two features) to 1.6 s (five features). For multi-core CPU usage, the training time increases from 79 s (two features) to 86 s (five features), whereas the prediction time per image increases from 3.8 s (two features) to 4.3 s (five features). Thereby, a small sacrifice in time performance can yield a significant improvement in the gradient boosting model through feature engineering by incorporating azimuth angle and/or pixel locations as input features for the training.

### 3.3. Diversity assessment

In this subsection, the gradient boosting model is employed to train various diverse datasets (Nickel, Battery-1, Battery-2, Battery-3 and Battery-4) collected under different experimental conditions as explained in Section 2.1. Three images are randomly selected from each dataset as the training set, and the rest of the images serve as a test set. Feature set C is applied as the features because a minor improvement was observed using feature set D as shown in Section 3.2.

**Figure 5:**
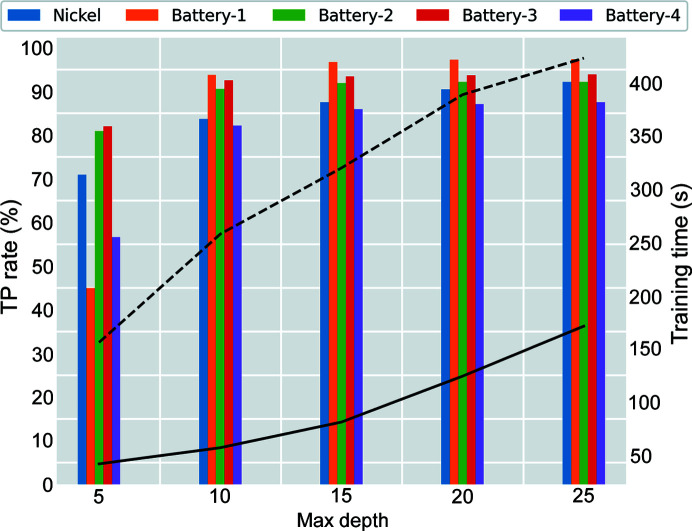
TP rate results for the datasets. The solid and dashed curves represent GPU and multi-core CPU training times as a function of gradient boosting tree depth.

Fig. 5 ▸ shows an increasing trend in TP rate as the maximum tree depth of the gradient boosting method increases. Significant increases are detected when the maximum depth increases from 5 to 10. These effects are especially prominent for the Battery-1 and Battery-4 datasets due to both datasets having an order of magnitude fewer positive labels in comparison with the rest of the datasets, exacerbating the class imbalance issue. The sharp improvements as the maximum depth increases show that the model needs more flexibility (*i.e.* increasing the maximum depth of the model) to accurately describe all datasets. On the other hand, the TP rates show negligible improvements after a maximum depth of 20 given the training dataset, where the Nickel dataset exhibits the highest increase in TP rate of 1.7%. Overall, the gradient boosting model effectively predicts the Nickel, Battery-1, Battery-2, Battery-3 and Battery-4 datasets with high accuracy at 92.2, 97.4, 92.2, 93.9 and 87.5%, respectively, at a maximum depth of 25. The TN rates are almost 100% on average for all datasets. The training time is illustrated by dashed and solid curves, representing the time measured with a multi-core CPU and with a GPU. Both training times increase as the maximum depth increases. However, the CPU training time increases at a slower rate whereas the GPU training time indicates an exponential increase. The prediction time stays constant at 1.4 s on average with the GPU. The GPU prediction run time is insensitive to the model depth because GPUs are optimized using massive parallelism with a high arithmetic intensity, and the *XGBoost* library supports GPU-accelerated capability (Mitchell & Frank, 2017 ▸). On the other hand, the CPU prediction time increases as the maximum depth increases with the following trend: 1.9, 2.9, 3.6, 4.0 and 4.5 s.

**Figure 6:**
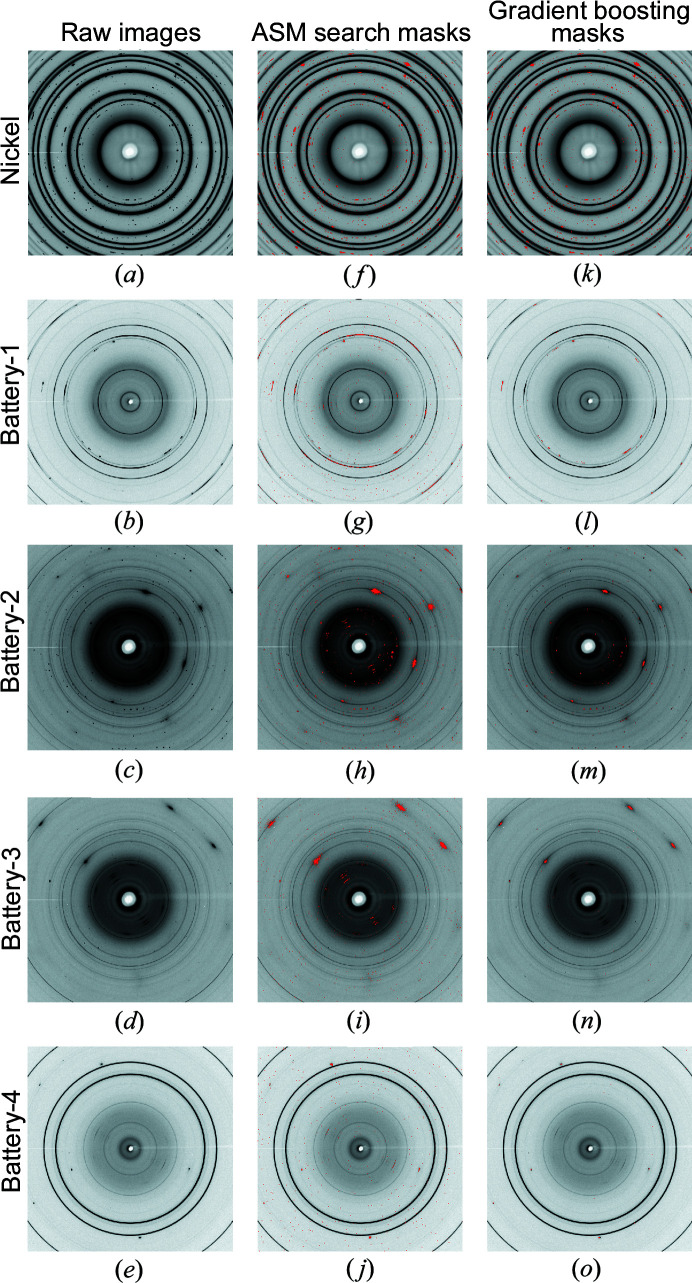
Qualitative masking results. (*a*)–(*e*) Raw images. (*f*)–(*j*) Results of masking using the ASM search. (*k*)–(*o*) Results of masking using the gradient boosting method. Each row consists of images that belong to the Nickel, Battery-1, Battery-2, Battery-3 or Battery-4 datasets, respectively, from top to bottom.

The qualitative masking results can be found in Fig. 6 ▸. Note that the Nickel image only consists of two diffraction characteristics: single-crystal diffraction spots and powder rings without texture. Accordingly, the ASM search can identify the signals, and the gradient boosting method also yields a reliable prediction. As for the rest of the images, the gradient boosting method identifies only the single-crystal diffraction spots without recognizing the preferred orientations, whereas the ASM search fails to differentiate between the preferred orientations or textures and single-crystal diffraction spots. The radii of the single-crystal diffraction-spot masks generated by the gradient boosting method are more tightly constrained than those generated by the ASM search approach. The phenomena can be clearly observed in the Battery-2 and Battery-3 images so that the gradient boosting masks have smaller radii compared with the ASM masks, in general. The circumstance can be caused by the skewed datasets, in which the images contain more negative labels than positive labels. Nevertheless, all of the single-crystal diffraction-spot centers are found by the gradient boosting method.

## 4. Conclusions and future work

In this work, the effectiveness and efficiency of ML methods such as SVM, KNN, extra trees, random forest and gradient boosting algorithms to identify and separate single-crystal diffraction spots in powder XRD images (containing more than 8 million pixels) in order to enable precise analyses of the 1D powder diffraction patterns, are investigated. It is shown that KNN, extra trees, random forest and gradient boosting display accurate predictions yielding >95% TP rate and 99.9% TN rate for the Nickel dataset. The gradient boosting method produces the best time and accuracy performances while offering both multi-core CPU and GPU support. The gradient boosting method has shown that it can reliably predict the XRD images with feature engineering, yielding TP rates in the range 87.5–97.4% when tested with all of the datasets. In addition, the TN rates remain at nearly 100% on average. The training time increases linearly as a function of training data size on both a multi-core CPU and a GPU, where the GPU training time increases at a slower pace than the multi-core CPU training time. It is also observed that the GPU prediction time of the gradient boosting method remains constant at 1.4 s despite the increase in model complexity (*i.e.* increasing maximum depth) and the size of the training dataset. The CPU prediction time experiences a slight increase as the model complexity increases, and it remains the same as training data size increases.

There are limitations to using the gradient boosting method for the identification and separation of single-crystal diffraction spots in XRD images. First, the XRD datasets are skewed representing class imbalance, in which the datasets have orders of magnitude more negative labels than the positive labels. As found in Fig. 6 ▸, the masks of the gradient boosting prediction exhibit smaller masking radii at single-crystal diffraction spots, yet it can better differentiate among artifacts and other characteristics in comparison with the ASM search approach. Second, extrapolation is a known drawback of ML methods. In this case, the gradient boosting method fails to identify the single-crystal diffraction spots in XRD images where the images have substantially stronger pixel intensities. A small subset of XRD images needs to be included in the model training if it has not been incorporated in the previous training as shown in Section 3.3.

We plan further investigations to enhance this ML-based artifact identification approach. The standard scaling normalization used in this paper confines the intensities to those found in the training dataset. We plan to explore other normalization techniques that do not bound the intensities to the training dataset. Additional feature engineering approaches such as using edge detection will be explored. Further, we plan to study the application of CNN to increase TP rate and transferability.

The application of ML methods, particularly the gradient boosting method, offers major improvements in both the effectiveness and efficiency relative to conventional and manual artifact identification methods. These ML methods offer substantial potential advancements in addressing the bottleneck in XRD artifact identification and reduce human intervention required during an experiment, opening the possibility of on-the-fly data processing capabilities. The code used in this study is available at https://github.com/AdvancedPhotonSource/AIRXD-ML-PUB.

## Figures and Tables

**Table 1:** Characteristics and experimental conditions of datasets SCD spots and POs are single-crystal diffraction spots and preferred orientations, respectively.

Dataset	No. of images	Characteristics	Experimental condition
Nickel	11	SCD spots	Temperature ramping
Battery-1	11	SCD spots and POs	Charging/discharging
Battery-2	12	SCD spots and textures	Charging/discharging
Battery-3	12	SCD spots and textures	Charging/discharging
Battery-4	12	SCD spots and textures	Charging/discharging

**Table 2:** Sets of features used for ML training

Feature set	No. of features	Features
Set A	2	Intensity and 2θ
Set B	3	Intensity, 2θ and azimuth
Set C	4	Intensity, 2θ and locations
Set D	5	Intensity, 2θ, azimuth and locations

**Table 3:** Optimal hyperparameters of the SVM, KNN, extra trees, random forest and gradient boosting methods Default values are used if they are not listed.

	Hyperparameters	Value
KNN	n_neighbors	3
	algorithm	auto
	leaf_size	5
	p	2
Extra trees	n_estimators	35
	criterion	gini
Random forest	n_estimators	35
	criterion	gini
	min_samples_split	2
Gradient boosting	n_estimators	35
	max_bin	10000
	min_child_weight	1
	max_depth	10

**Table 4:** Benchmarking results using the Nickel dataset TN and TP rates represent the true negative and true positive rates evaluated with the Nickel test set. The training/testing time performances are measured with CPU(GPU).

Algorithm	TN rate (%)	TP rate (%)	Time (s)
SVM	78.45 ± 0.23	46.17 ± 3.31	2400/1500
KNN	99.93 ± 0.13	94.69 ± 1.01	220/42
Extra trees	99.88 ± 0.17	97.73 ± 0.68	260/4.8
Random forest	99.96 ± 0.06	97.33 ± 0.30	500/4.5
Gradient boosting	99.94 ± 0.10	98.22 ± 0.39	43(11)/2.2(0.94)

**Table 5:** Overall predicted TP rates in percentage of Nickel and Battery-1 datasets against varying feature sets The numbers in parentheses represent the number of features for each set.

Dataset	Set A (2)	Set B (3)	Set C (4)	Set D (5)
Nickel	95.2	96.2	98.1	98.9
Battery-1	54.8	85.4	97.5	98.0
